# Supplementary material for: Asynchronous magnetic resonance elastography: Shear wave speed reconstruction using noise correlation of incoherent waves
Source: Magn Reson Med. 2022 Oct 27;89(3):990–1001. doi: 10.1002/mrm.29502 (PMC9792433; doi:10.1002/mrm.29502)
Supplement: Supplementary file 1 — DATA S1 MATLAB code used to generate the numerical simulations of Figure 1 [file MRM-89-990-s002.zip › k-Wave/helpfiles/reorderSensorData.html]

reorderSensorData :: Functions (k-Wave)


# reorderSensorData

Reorder sensor data from kspaceFirstOrder2D based on angle.

## Syntax

```
[reordered_sensor_data, indices_new] = reorderSensorData(kgrid, sensor, sensor_data)
```

## Description

`reorderSensorData` reorders the time series from `kspaceFirstOrder2D` based on the angle that each sensor point makes with the centre of the grid. The sensor mask must be a binary mask, and the angles are defined from the upper left quadrant or negative y-axis in the same way as within `makeCircle` and `makeCartCircle`.

## Inputs

|  |  |
| --- | --- |
| `kgrid` | k-Wave grid object returned by `kWaveGrid` |
| `sensor` | k-Wave sensor structure where `sensor.mask` is defined as a binary grid |
| `sensor_data` | sensor data returned by `kspaceFirstOrder2D` ordered using MATLAB's standard column-wise linear matrix indexing |

## Outputs

|  |  |
| --- | --- |
| `reordered_sensor_data` | time varying sensor data reordered by the angle that each sensor point makes with the centre of the grid |
| `indices_new` | the indices of the reordered time series, where `reordered_sensor_data = sensor_data(indices_new, :)` |

## See Also

`kspaceFirstOrder2D`, `reorderBinarySensorData`, `unmaskSensorData`
